# Key Gaps in the Prevention and Treatment of Obesity in Children and Adolescents: A Critical Appraisal of Clinical Guidelines

**DOI:** 10.3390/children12030347

**Published:** 2025-03-10

**Authors:** Francesca Filippi-Arriaga, Michael Georgoulis, Eirini Bathrellou, Meropi D. Kontogianni, Eduard Mogas, Graciela Gastelum, Andreea Ciudin

**Affiliations:** 1 Endocrinology and Nutrition Department, Obesity Unit, Vall Hebron University Hospital, 08035 Barcelona, Spain; francesca.filippi@vhir.org (F.F.-A.); graciela.gastelum@vhir.org (G.G.); 2 Department of Nutrition and Dietetics, School of Health Sciences and Education, Harokopio University of Athens, 17671 Athens, Greece; mgeor@hua.gr (M.G.); ebathrellou@hua.gr (E.B.); mkont@hua.gr (M.D.K.); 3 Pediatric Endocrinology Department, Vall Hebron University Hospital, 08035 Barcelona, Spain; eduard.mogas@vallhebron.cat

**Keywords:** obesity, children, adolescents, prevention, treatment, clinical guidelines

## Abstract

Background: The worldwide increase in the prevalence of childhood obesity necessitates effective prevention and treatment strategies. Clinical practice guidelines (CPGs) offer guidance, but significant heterogeneity or lack of practical application exists in their recommendations. The purpose of the present study is to provide an expert, comprehensive, and comparative analysis of gaps in current CPGs for the prevention and treatment of obesity in children and adolescents. Results: A total of 14 CPGs were identified, focusing on childhood obesity prevention (n = 11), treatment (n = 9), or both (n = 6). Prevention CPGs generally recommend body mass index (BMI) assessment, but specific measurement frequency is often absent. While some provide age-specific dietary recommendations, graphical tools are lacking. Recommendations for increased physical activity and reduced screen time are common, but age-stratified guidance is deficient. Furthermore, recommendations regarding mental health and sleep are notably absent. Treatment CPGs utilize BMI for obesity diagnosis, but inconsistencies in cut-off points persist. Comorbidity assessment is generally recommended, yet age-specific guidance remains lacking. Dietary recommendations are present in most CPGs, but many lack detailed specifications (e.g., meal frequency, portion sizes, macronutrient distribution, age-appropriate examples). Most CPGs advocate for 60 min of daily physical activity and limit screen time to 2 h per day. Recommendations concerning sleep are consistently absent. While parental involvement is acknowledged, specific guidance for active participation in prevention and treatment is deficient. Pharmacological treatment options are frequently outdated, and surgical intervention is reserved for exceptional cases of severe obesity. Conclusions: Standardizing BMI cut-off points and defining age groups across CPGs would improve consistency and comparability in the diagnosis, prevention, and treatment of childhood obesity. Tailoring recommendations for diet, physical activity, sedentary behavior, and sleep to specific age groups would ensure developmentally appropriate interventions. A stronger emphasis on early prevention strategies is needed to address the root causes of obesity. Clear guidance for parents and families would facilitate their active engagement in prevention and treatment. Up-to-date information regarding pharmacological and surgical treatments is imperative.

## 1. Introduction

The global prevalence of overweight and obesity among children and adolescents has exhibited a concerning escalation [1,2,3]. Pertinent data from the World Health Organization (WHO) reveal that the prevalence of overweight and obesity among children and adolescents has reached alarming levels, with over 390 million young people affected in 2022 [4]. This surge in prevalence highlights the urgent need for effective prevention and treatment strategies to mitigate the risk of developing associated complications and comorbidities, as well as managing existing conditions. The etiology of childhood obesity is complex and multifactorial, encompassing a range of factors including genetic predisposition, nutritional habits, physical activity levels, comorbidities, sleep patterns, and familial and community environments [3,5,6]. This intricate interplay of factors necessitates a comprehensive understanding by healthcare professionals, families, and patients alike. To navigate this complexity, clinical practice guidelines (CPGs) are sought after to inform both preventative strategies and treatment modalities. However, despite the availability of CPGs, considerable heterogeneity can exists in their recommendations, raising concerns about their practicality and implementation across diverse healthcare systems, primary and specialized care settings, and within the family dynamic [7]. This narrative review aims to provide an expert, comprehensive, and comparative analysis of gaps in current CPGs for the prevention and treatment of obesity in children and adolescents.

## 2. Materials and Methods

A comprehensive search was executed in PubMed, Scopus, and Google Scholar databases to identify CPGs for the prevention and/or treatment of obesity in children and adolescents aged 2 to 19 years. The search parameters encompassed all publications indexed up to January 2025, without temporal restrictions on prior studies. Search terms employed included “clinical guideline”, “society”, “obesity”, “overweight”, “children”, and “adolescents”. To be included in this review, CPGs had to meet specific criteria: publication in English, origin from Western regions (Europe, the United States of America (USA), Canada, or Oceania), and development or publication by a national or international scientific society or organization. This geographic focus allows for a comparative analysis of guidelines within regions sharing similar socioeconomic development and cultural heritage rooted in European traditions. CPGs failing to meet these criteria, including documents lacking clinical recommendations and those available exclusively in languages other than English, were excluded. Any relevant documents identified outside the initial search were added in a targeted manner. Two investigators (F.-F.A. and A.C.) independently conducted document selection based on predefined inclusion and exclusion criteria. Documents were categorized as clinical guidelines for prevention of obesity in children and adolescents, treatment of obesity in children and adolescents, or both. Discrepancies regarding document inclusion were resolved through in-depth content review and consensus. No direct contact was made with authors or organizations. Two investigators (F.-F.A. and A.C.) performed data extraction and synthesis. There were 14 different CPGs included (Table 1). The content of the selected CPGs was analyzed separately for prevention and treatment recommendations. CPGs were classified as focusing on prevention, treatment, or both aspects. The information extracted included the assessment of obesity (weight status, health status), lifestyle recommendations (diet, physical activity, sedentary behavior, and sleep), treatment (drugs, surgery), and the role of parents/guardians in the prevention/treatment of childhood obesity (detailed information is provided in Supplementary Materials).

## 3. Results

The CPGs presented substantial heterogeneity in their recommendations, with some focusing on prevention (n = 11) [8,9,10,11,12,13,14,15,16,17,18], others on treatment (n = 9) [5,8,9,10,13,15,17,19,20], and most addressing both aspects (n = 6) [8,9,13,15,17,19].

### 3.1. Clinical Guidelines for the Prevention of Obesity in Children and Adolescents

Concerning the evaluation of weight status, the majority of clinical practice guidelines (CPGs) for the prevention of obesity in children and adolescents advocated for the utilization of body mass index (BMI) measurement; however, a substantial proportion failed to specify recommendations regarding the frequency of assessment [8,9,11,14,17,18]. With respect to health status evaluation, most CPGs for prevention did not include the assessment or screening of organic pathologies (genetic disorders, endocrine disorders) that could lead to the development of obesity [8,11,12,13,16,18]. Furthermore, obesity in children and adolescents can be associated with mental health disorders; however, specific recommendations for their assessment were notably absent from prevention-oriented guidelines [8,9,10,11,12,13,14,15,16,17,18].

Within the domain of lifestyle modifications, several clinical practice guidelines (CPGs) advocate for exclusive breastfeeding for at least the first 6 months of life, as a prophylactic measure against subsequent obesity development [8,9,11]. Furthermore, all CPGs incorporate general recommendations pertaining to a varied and healthy diet, and most of them mention the importance of schools in fostering the adoption of healthy dietary habits. However, a subset of CPGs are more detailed regarding optimal dietary intake by providing specific portion recommendations tailored to different age groups [11,16,17]. Notably, certain guidelines emphasize the importance of incorporating high-fiber foods, such as fruits, vegetables, and whole grains [11,14,16]. For example, the Registered Nurses Association of Ontario (RNAO) guidelines present a table detailing that children aged 4–8 should have five servings of vegetables and fruit, four servings of grain products, and three servings of milk and alternatives daily. In contrast, children aged 9–13 should have six servings of vegetables and fruit, six servings of grain products, and three servings of milk and alternatives This age-specific breakdown ensures that dietary recommendations are commensurate with the evolving nutritional requirements of children during growth and development [11]. Additionally, the AND’s (Academy of Nutrition and Dietetics) guide underscores the critical role of nutritionists in obesity, recognizing their unique qualifications to provide nutrition counseling in pediatric settings [18]. Regarding graphical tools and visual aids, most CPGs fail to include diagrams or tables that could exemplify appropriate servings of different food groups and translate dietary recommendations to meaningful counselling guidance [13,15,16,17,18,19].

Physical activity is a determinant factor in pediatric cardiovascular, musculoskeletal, and mental health, as well as in physical, social, and cognitive development [21]. Most CPGs oriented on prevention concur on the recommendation of a minimum of 60 min of structured physical activity daily [11,13,15,16,17]. The majority of CPGs highlight the importance of schools to promote physical education and sporting activities [5,8,9,12]. However, most of the CPGs lack clear recommendations regarding age-specific exercise duration and intensity. Additionally, they fail to provide examples of developmentally appropriate activities and sports for different age groups [8,9,12,14,18]. Mitigation of sedentary behavior is a critical prophylactic strategy against obesity, and interventions aimed at reducing recreational screen media exposure may result in a substantial increase in children’s engagement in physical activity [22]. Most of the reviewed CPGs advocate for a restriction of screen time (e.g., television viewing, video gaming) to a maximum of 2 h per day for children exceeding 2 years of age, with complete abstinence recommended for younger children [8,11,15,16]. Nevertheless, certain preventive CPG recommendations lack sufficient information on screen use and time limits [5,12,13,14]. Moreover, it is unclear whether these recommendations should be universal or adjusted to specific age groups. Multiple cross-sectional investigations have established a correlation between reduced sleep duration and disrupted sleep cycles with the development of obesity in children [23,24,25,26]. Interestingly, there is lack of recommendations on sleep habits as this domain is largely omitted from the majority of prevention CPGs [5,8,9,11,12,13,14,16,17,18,19,20].

### 3.2. Clinical Guidelines for the Treatment of Obesity in Children and Adolescents

Nine of the guidelines identified were oriented on obesity treatment [2,3,4,5,8,10,12,13,14]. All CPGs advocate for the assessment of weight status utilizing BMI percentiles for the diagnosis of overweight and obesity. For children ≥2 years of age, most CPGs agree on the diagnosis of overweight if the BMI is ≥85th percentile and <95th percentile for age and sex, and for the diagnosis of obesity if BMI is ≥95th percentile for age and sex [8,10,15]. However, certain CPGs employ alternative percentile thresholds for diagnostic classification [9,15,19]. For instance, the Spanish Clinical Practice Guideline for the Prevention and Treatment of Childhood and Juvenile Obesity designates a BMI ≥ 97th percentile for age and sex as the diagnostic criterion for obesity [9]. Similarly, the Scottish Intercollegiate Guidelines Network recommend that for clinical use, obese children are those with a BMI ≥98th percentile of the United Kingdom (UK) 1990 reference chart for age and sex (see Supplementary Materials) [19].

Most of the treatment CPGs advocate for a comprehensive medical history and a thorough physical examination to exclude secondary etiologies of obesity in pediatric populations [8,9,19,20]. For the assessment of health status, most CPGs concur that children with obesity should be evaluated for comorbidities [10,15,17,19,20]. This typically includes the measurement of blood pressure, blood glucose, and lipid profile [10,15,17,20]. Regarding comorbidity assessment, an optimal approach would involve the delineation of specific indications and diagnostic studies tailored to distinct age groups [13,15,20]. A salient example of this age-stratified approach is provided by the Italian guideline for Diagnosis, treatment, and prevention of pediatric obesity suggests [15]. This CPG includes the following recommendations: measuring cholesterol, high-density lipoprotein cholesterol (HDL), and triglycerides in all children and adolescents with obesity starting at the age of 6; measuring blood pressure in all children with overweight or obesity starting at the age of 3; measuring fasting blood glucose in all children and adolescents with overweight or obesity starting at the age of 6, as the first step for screening prediabetes and type 2 diabetes; and assessing transaminases and liver ultrasound in all children and adolescents with obesity starting at the age of 6, among others [15].

When addressing mental health, CPGs acknowledge the importance of psychological support for the treatment of overweight and obesity in children and adolescents, and they advocate for referral to specialists in the presence of suspected depressive and/or anxious symptoms, dysmorphophobia traits, suicidal risk, and eating disorders [8,13,15,19]. However, they do not recommend a psychological routine or follow-up evaluation.

Dietary recommendations within treatment-oriented CPGs frequently encompass general guidance promoting the consumption of a balanced and varied diet, negative caloric balance, eating at regular times, and increasing the intake of fruit and vegetables [8,9,13,15,20]. Regarding meal patterns, some examples of specific recommendations include a consistent meal schedule with five structured meals per day at regular intervals, minimizing snacking and avoiding skipping meals [17]. Regarding dietary patterns, CPGs emphasize balanced nutrition by including protein, carbohydrates, and healthy fats in every meal, in the consumption of fruits, vegetables, fiber-rich cereals, and low-fat dairy products, as well as the limitation of high-energy and low-nutrient foods, such as sweetened drinks, fast food, sugary snacks, and sauces [9,13,20]. Additional recommendations include the prioritization of water consumption over sugar-sweetened beverages, as well as the promotion of mindful eating practices, such as food label scrutiny, family meal encouragement, limitation of restaurant dining, and age-appropriate portion control [5,15,17].

In the realm of physical activity, most treatment-oriented clinical practice guidelines (CPGs) align with their prevention counterparts, recommending a minimum of 60 min of structured physical activity per day [10,13,15,17,19,20]. Concerning sedentary behavior, most CPGs offer general guidance on curtailing time spent in sedentary activities, such as television viewing, video game playing, and internet use [8,9,15,19]. Treatment CPGs also concur with prevention CPGs in advocating for the restriction of screen time to 1–2 h per day [9,13,17,19,20]. A consistent deficiency across all treatment CPGs is the lack of specific recommendations pertaining to adequate sleep duration and quality [5,8,9,10,13,15,17,19,20].

Regarding pharmacological interventions, a substantial proportion of CPGs exhibit outdated recommendations, suggesting medications such as sibutramine, liraglutide, or orlistat, without providing age-stratified guidance [9,17,19]. Most CPGs concur that weight-loss pharmacotherapy should be reserved for adolescents ≥12 years of age with obesity (BMI ≥ 95th percentile), in accordance with medication indications, risk–benefit profiles, and as an adjunct to behavioral and lifestyle interventions [9,17,20]. Some CPGs indicate that bariatric surgery in adolescents is recommended only in extreme cases of severe obesity (BMI ≥ 40 kg/m^2^) with significant comorbidities or extreme obesity (BMI ≥ 50 kg/m^2^) when intensive lifestyle interventions, with or without drug treatment, have failed for at least six months [8,9,13,17]. Eligibility criteria typically include attainment of Tanner stage 4 or 5 pubertal development and near-final or final adult height. Furthermore, CPGs emphasize that bariatric surgery should be performed exclusively in centers with multidisciplinary teams experienced in adolescent bariatric surgery [9,13,15,17]. The guidelines emphasize that surgery represents the last resort and should only be considered after all other treatment options have been exhausted.

### 3.3. Parental Role

Research has shown that involving parents or caregivers in the prevention and treatment of children and adolescents with obesity leads to better long-term weight management compared to focusing solely on the child without parental involvement [24,25]. Most CPGs generally mention or encourage behavioral interventions incorporating parental and/or familial involvement and acknowledge the pivotal role of the family in fostering a lifestyle conducive to obesity prevention. However, they do not include specific recommendations (diet, sleep, physical activity, family environment) that parents should follow to prevent or treat obesity from home [5,8,11,12,13,15,19].

**Table 1:** Comparison of clinical practice guidelines for the prevention and treatment of obesity in children and adolescents.

Guideline	Assessment				Lifestyle Changes			Treatment		Parental Role
CPGs Focused on Prevention	Weight Status/Diagnosis	Health status	Mental Health	Diet	Physical Activity	Sedentary Time	Sleep	Drugs	Surgery	
Canadian clinical practice guidelines on the management and prevention of obesity in adults and children [summary] (2007) [8].	**✔**	**✔**	**X**	**✔**	**✔**	**✔**	**X**	**-**	**-**	**✔**
Spain. Clinical Practice Guideline for the Prevention and Treatment of Childhood and Juvenile Obesity (2009) [9].	**✔**	**✔**	**X**	**✔**	**✔**	**✔**	**X**	**-**	**-**	**✔**
Scottish Intercollegiate Guidelines Network: Management of Obesity A national clinical guideline (2010) [19].	**✔**	**✔**	**X**	**X**	**X**	**X**	**X**	**-**	**-**	**✔**
Canada. Primary Prevention of Childhood Obesity (2nd edition). International Affairs and Best Practice Guidelines. Registered Nurses Association of Ontario (RNAO) (2014) [11].	**✔**	**✔**	**X**	**✔**	**✔**	**✔**	**X**	**-**	**-**	**✔**
England. National Institute for Health and Care Excellence (NICE). Obesity prevention clinical guideline (2015) [12].	**X**	**X**	**X**	**✔**	**✔**	**X**	**X**	**-**	**-**	**✔**
European Society of Endocrinology and the Pediatric Endocrine Society (2017) [13].	**✔**	**X**	**X**	**✔**	**✔**	**✔**	**✔**	**-**	**-**	**✔**
United States. Screening for Obesity in Children and Adolescents: US Preventive Services Task Force Recommendation Statement (2017) [14].	**✔**	**✔**	**X**	**✔**	**✔**	**✔**	**✔**	**-**	**-**	**X**
Italy. Diagnosis, treatment and prevention of pediatric obesity: Consensus position statement of the Italian Society for Pediatric Endocrinology and Diabetology and the Italian Society of Pediatrics (2018) [15].	**X**	**✔**	**X**	**✔**	**✔**	**✔**	**✔**	**-**	**-**	**X**
Germany. Current Guidelines for Obesity Prevention in Childhood and Adolescence (2018) [16].	**X**	**X**	**X**	**✔**	**✔**	**✔**	**X**	**-**	**-**	**X**
Poland. Childhood Obesity: Position Statement of the Polish Society of Pediatrics, Polish Society for Pediatric Obesity, Polish Society of Pediatric Endocrinology and Diabetes, the College of Family Physicians in Poland, and the Polish Association for Study on Obesity (2022) [17].	**✔**	**✔**	**X**	**✔**	**✔**	**✔**	**X**	**-**	**-**	**✔**
United States. Prevention of Pediatric Overweight and Obesity: Position of the Academy of Nutrition and Dietetics Based on an Umbrella Review of Systematic Reviews (2022) [18].	**✔**	**X**	**X**	**✔**	**✔**	**✔**	**X**	**-**	**-**	**✔**
**CPGs Focused on Treatment**	**Weight status/diagnosis**	**Health Status**	**Mental** **Health**	**Diet**	**Physical Activity**	**Sedentary Time**	**Sleep**	**Pharmacotherapy**	**Surgery**	**Parental Role**
Canadian clinical practice guidelines on the management and prevention of obesity in adults and children [summary] (2007) [8].	**✔**	**✔**	**✔**	**✔**	**✔**	**✔**	**X**	**✔**	**✔**	**X**
Spain. Clinical Practice Guideline for the Prevention and Treatment of Childhood and Juvenile Obesity (2009) [9].	**✔**	**✔**	**✔**	**✔**	**✔**	**✔**	**X**	**✔**	**✔**	**✔**
Scottish Intercollegiate Guidelines Network: Management of Obesity A national clinical guideline (2010) [19].	**✔**	**✔**	**✔**	**✔**	**✔**	**✔**	**X**	**✔**	**✔**	**✔**
European Society of Endocrinology and the Pediatric Endocrine Society (2017) [8]	**✔**	**✔**	**✔**	**✔**	**✔**	**✔**	**X**	**✔**	**✔**	**X**
Italy. Diagnosis, treatment and prevention of pediatric obesity: consensus position statement of the Italian Society for Pediatric Endocrinology and Diabetology and the Italian Society of Pediatrics (2018) [15].	**✔**	**✔**	**✔**	**✔**	**✔**	**✔**	**X**	**✔**	**✔**	**✔**
Poland. Childhood Obesity: Position Statement of the Polish Society of Pediatrics, Polish Society for Pediatric Obesity, Polish Society of Pediatric Endocrinology and Diabetes, the College of Family Physicians in Poland, and the Polish Association for Study on Obesity (2022) [17].	**✔**	**✔**	**✔**	**✔**	**✔**	**✔**	**X**	**✔**	**✔**	**✔**
United States. Treatment of Pediatric Overweight and Obesity: Position of the Academy of Nutrition and Dietetics Based on an Umbrella Review of Systematic Reviews (2022) [5].	**✔**	**X**	**✔**	**✔**	**X**	**X**	**X**	**X**	**X**	**✔**
American Academy of Pediatrics. Executive Summary: Clinical Practice Guideline for the Evaluation and Treatment of Children and Adolescents with Obesity (2023) [20].	**✔**	**✔**	**✔**	**✔**	**✔**	**✔**	**X**	**✔**	**✔**	**X**
Italy. Cardiometabolic risk in children and adolescents with obesity: a position paper of the Italian Society for Pediatric Endocrinology and Diabetology (2024) [10].	**✔**	**✔**	**X**	**✔**	**✔**	**✔**	**X**	**X**	**X**	**X**

Guidelines that addressed specific or general aspects (**✔**); guidelines that did not address specific or general aspects (X). CPGs: clinical practice guidelines.

## 4. Discussion

Obesity in children and adolescents is a significant public health concern worldwide. To address this issue, CPGs for the prevention and treatment of obesity in children and adolescents have been developed. However, these CPGs are very heterogenous between European countries and worldwide and present several gaps and challenges. Heterogenicity in CPGs often leads to inconsistencies in recommendations and confusion to healthcare providers, patients, and parents [7]. Based on the gaps and heterogeneity identified in the present review, we have created a proposal of recommendations to cover gaps in future CPGs for the prevention and treatment of obesity in children and adolescents (see Table 2).

Specifically, while most guidelines use the same BMI cut-off points to diagnose overweight (≥85th percentile, <95th percentile for age and sex) and obesity (≥95th percentile for age and sex) [8,15], some utilize different cut-off points [9,15,19]. These discrepancies in diagnostic thresholds pose challenges in several areas, including accurately diagnosing obesity, conducting clinical studies with comparable results, managing data consistently, and unifying information across European countries and global healthcare systems. To address these challenges, the standardization of BMI cut-off points across European and global healthcare systems is imperative. Furthermore, the development and standardization of adipose-tissue-focused diagnostic and stratification tools are urgently needed. Recommendations for the periodic assessment of BMI, such as annual calculation and plotting of BMI percentiles during well-child or sick-child visits, or monthly measurement of body weight, may be of help to propose in future guidelines as an adequate periodicity for the measurement of these parameters [13].

Another critical gap identified in the current CPGs is the lack of an age-specific approach to recommendations. This is particularly crucial in pediatric populations due to their dynamic physiology, which influences nutritional requirements, medication responses, and engagement in physical activity. To address this, a global effort to define and unify age group classifications is necessary. This will facilitate the development of targeted, age-appropriate recommendations for assessment and intervention in the domains of diet, physical activity, sedentary behavior, screen time, and sleep. The current review also identified a limited focus on prevention [8,9,10,11,12,13,14,15,16,17].

A substantial proportion of current clinical practice guidelines (CPGs) prioritize the management of established obesity, rather than emphasizing primordial and primary prevention strategies [5,8,9,10,13,15,17,19,20]. Early intervention is crucial for addressing the root causes of overweight and obesity. It is important to consider the expert support of nutritionists in providing nutritional counseling and guidance [18]. The role of nutritionists is crucial in preventing obesity in children, as they can provide tailored guidance and interventions to promote healthy eating habits and address individual nutritional needs [18].

Furthermore, the integration of mental health assessment and support is essential. This includes the implementation of screening protocols to identify mental health disorders and facilitate prompt referral to specialists in the presence of suspected depressive or anxious symptoms, dysmorphophobia, suicidal ideation, or eating disorders. The establishment of standardized protocols for the monitoring of psychological symptoms is also crucial. For example, the American Academy of Pediatrics Clinical Practice Guideline for the Evaluation and Treatment of Children and Adolescents With Obesity recommends monitoring for symptoms of depression in children and adolescents with obesity and conducting annual evaluations for depression in adolescents 12 y and older with a formal self-report tool [20].

In addition, it is appropriate that the guidelines include parameters on adequate sleep duration and quality of sleep according to age. An example is the sleep recommendations of the Italian Guideline for Diagnosis, Treatment and Prevention of Pediatric Obesity (from 4–12 months: 12–16 h/day; 1–2 years: 11–14 h/day; 3–5 years: 10–13 h/day; 6–12 years: 9–12 h/day; 13–18 years: 8–10 h/day) [15]. Probably the lack of relevant published data has not allowed thus far the provision of robust recommendations regarding sleep duration/quality/habits for the prevention and treatment of obesity.

Compared to other aspects of obesity management, such as physical activity or mental health, dietary recommendations appear to be more comprehensively addressed in the CPGs. While variability exists at the level of detail and specificity, most CPGs provide guidance on both the prevention and treatment of obesity through dietary interventions. This suggests that there is a greater consensus and understanding of the role of diet in children and adolescent’s obesity management compared to other areas, where recommendations may be more inconsistent or lacking in detail. As the most important tool in obesity prevention and treatment, CPGs prioritize providing guidance on dietary interventions. However, some CPGs still lack an age-specific approach regarding portion sizes, quantities, or percentages of macronutrients [8,9,19]. Guidelines should include helpful visuals, such as diagrams and tables, for practitioners, patients, and families. These visuals can illustrate daily servings and provide examples of portion sizes for vegetables, fruits, grains, dairy, and meat [11].

Regarding physical activity, an ideal approach would be to specify recommended exercise duration and intensity levels tailored to each age group. Additionally, providing examples of age-appropriate sports and activities would offer practical support in promoting physical activity. This review reveals that physical activity recommendations are often homogenous and do not differentiate across the domains of obesity prevention or treatment, nor across different age groups. The majority of CPGs fail to provide examples of developmentally appropriate activities and sports for different age groups [8,9,12,14]. This omission of information hinders health service providers and parents in their efforts to promote optimal physical activity and development in children and adolescents. A more effective approach is exemplified by the Germany Current Guidelines for Obesity Prevention in Childhood and Adolescence, which recommend that children aged 3–5 years engage in a minimum of 60 min of structured physical activity daily, in addition to 60 min to several hours of unstructured physical activity, with inactivity limited to less than 60 min at a time (excluding sleep) [16]. This CPGs also recommends that school-aged children and adolescents engage in a minimum of 60 and 90 min of moderate to intense physical activity daily, respectively, or achieve at least 10,000 steps per day [16].

Despite obesity being a chronic disease, CPGs often lack explicit recommendations for long-term follow-up care, which is crucial for sustained weight management and overall health optimization. Furthermore, guidance on obesity screening frequency is often ambiguous. Clear and concise recommendations regarding screening and monitoring protocols for pediatric patients with obesity are essential to maximize treatment adherence and optimize health outcomes.

Concerning pharmacological and surgical interventions, CPGs appropriately designate these modalities as last-resort options for individuals with severe comorbidities refractory to lifestyle modifications. However, CPGs should be updated to reflect current scientific evidence regarding pharmacological treatment options. Bariatric surgery remains a last resort for adolescents with severe obesity (BMI ≥ 40 kg/m^2^) and significant comorbidities, or extreme obesity (BMI ≥ 50 kg/m^2^), and should be performed exclusively in specialized centers with multidisciplinary teams experienced in adolescent bariatric surgery.

Although clinical practice guidelines (CPGs) frequently advocate for parental involvement in the management of pediatric obesity, specific guidance for parents is often lacking. Examples of actionable recommendations for parents include prioritizing breastfeeding recommendations for infants [8,9], enriching their diet with nutritious foods, and structuring meals according to healthy eating principles [11,16,17]. Also, parents should ensure that children engage in at least 60 min of moderate-to-vigorous physical activity per day, incorporating age-appropriate activities and play [13,15,17,19,20]. If necessary, parents should consider family-wide lifestyle changes in diet and exercise to manage weight concerns collectively. Additionally, actively engaging in preventative healthcare and creating a home environment that supports healthy choices are crucial for promoting children overall well-being [17]. Finally, to highlight the crucial role of parents in preventing and treating children and adolescent’s obesity, the guidelines should not only elucidate the scientific rationale for parental involvement but also incorporate a dedicated section outlining specific, actionable steps for parents to implement. This will empower parents to actively participate in their child’s health management journey and contribute to improved health outcomes.

## 5. Conclusions

Addressing the gaps and inconsistencies of current clinical practice guidelines (CPGs) is essential for developing more effective, practical, and evidence-based CPGs in Europe and worldwide. A crucial step toward enhancing the consistency and comparability of childhood obesity management is the standardization of body mass index (BMI) cut-off points for diagnostic purposes. Furthermore, the exploration and integration of alternative diagnostic modalities that consider adipose tissue distribution and functionality are warranted.

Clinical practice guidelines (CPGs) must comprehensively address all aspects of obesity management, including health status, diet, physical activity, screen time, sleep, and treatment options. Crucially, recommendations within these domains must be stratified by age group to ensure developmental appropriateness and optimize intervention efficacy. This age-specific approach should be applied across all aspects of assessment and intervention.

A strong emphasis on early prevention strategies is needed to address the root causes of obesity, while providing clear guidance to parents and families would facilitate their active involvement in both prevention and treatment. Up-to-date information regarding pharmacological and surgical interventions is needed.

## 6. Limitations

This study has certain limitations. First, it excluded clinical practice guidelines (CPGs) from Latin American countries due to differing socioeconomic contexts and potential cultural influences on CPG development and implementation. However, obesity in children and adolescents is a significant public health concern in Latin America, warranting future research on region-specific prevention and treatment challenges and opportunities. Second, the CPGs included were developed by various organizations and societies using different methodologies and evidence bases, potentially contributing to the observed variability in recommendations. Third, the study focused on analyzing CPG content but did not assess their actual implementation or impact on clinical practice. While this was not within the scope of our review, future research may evaluate the real-world effectiveness of these guidelines in promoting the prevention and treatment of obesity in children and adolescents.

## 7. Future Perspectives

The review of the scientific evidence of CPGs was not within the scope of the present work. However, it is important to conduct a thorough updated review of the scientific evidence to develop more precise and targeted recommendations for preventing and treating obesity in children and adolescents in future CPGs. This review should include an examination of the available evidence on various aspects, including obesity management, health status, diet, physical activity, screen time, and treatment. Additionally, the review should address the lack of robust recommendations regarding sleep duration, quality, and habits for the prevention and treatment of obesity.

## Figures and Tables

**Table 2:** Recommendations to cover gaps in future CPGs for the prevention and treatment of obesity in children and adolescents.

Areas	Recommendations
Weight status	Create universal diagnostic parameters for obesity in children and adolescents Same BMI percentiles Exploration of adipose-tissue-focused diagnostics
Health status	Stratification in age groups Instructions for evaluation of comorbidities Description of complementary tests to perform Indicate frequency of follow-up
Mental health	Indicate who to screen Indicate when to screen Describe what tests to use (e.g., questionnaires) Indicate frequency of follow-up
Diet	Stratification in age groups Describe number of meals per day Describe portion sizes Include macronutrient percentages Describe types of foods to avoid Provide graphic examples Indicate frequency of follow-up
Physical activity	Stratification in age groups Indicate type of physical activity Indicate duration of physical activity Indicate intensity of physical activity Differentiate between prevention and treatment recommendations if possible Provide examples of routine
Sedentary time	Stratification in age groups Indicate duration of screen time Recommendations on screen-free times Describe screens habits to avoid (e.g., screen during meals) Differentiate between prevention and treatment recommendations if possible
Sleep	Stratification in age groups Indicate duration of sleep Indicate specific sleep hygiene behaviors Indicate regular sleep schedule recommendations Differentiate between prevention and treatment recommendations if possible
Pharmacotherapy	Include updated pharmacological treatment options Indicate specific indications according to age and comorbidities Indicate frequency of follow-up
Surgery	Include specific indications according to age, BMI, tanner, and comorbidities Indicate frequency of follow-up
Parental role	Specific recommendations for parents regarding their children: Nutrition Physical activity Sleep Screen time Participation in their children’s medical appointments

BMI: body mass index.

## Data Availability

Data is contained within the article or Supplementary Materials. Further inquiries can be directed to the corresponding author.

## Supplementary Materials

The following supporting information can be downloaded at https://www.mdpi.com/article/10.3390/children12030347/s1. Includes a comparative table with the specific information found of each CPG reviewed. It includes the assessment of obesity (weight status, health status), lifestyle recommendations (diet, physical activity, sedentary behavior, and sleep), treatment (drugs, surgery), and the role of parents/guardians in the prevention/treatment of childhood obesity.

## Abbreviations

The following abbreviations are used in this manuscript:

BMI	body mass index
CPGs	clinical practice guidelines
HDL	high-density lipoprotein cholesterol
RNAO	Registered Nurses Association of Ontario
USA	United States of America
